# Procalcitonin: A promising tool or just another overhyped test?

**DOI:** 10.7150/ijms.39367

**Published:** 2020-01-18

**Authors:** Robin Paudel, Prerna Dogra, Ashley A Montgomery-Yates, Angel Coz Yataco

**Affiliations:** 1 Division of Pulmonary and Critical Care, University of Kentucky, Lexington, Kentucky;; 2 Division of Hospital Medicine, University of Kentucky, Lexington, Kentucky;; 3 Lexington Veterans Affairs Medical Center, Lexington, Kentucky;; 4 University of Kentucky College of Medicine

## Abstract

Sepsis is the leading cause of death worldwide. Timely administration of antibiotics is recognized as the cornerstone in the management of sepsis. However, inappropriate use of antibiotics may lead to adverse effects and the selection of drug-resistant pathogens. Microbiological cultures remain the gold standard to diagnose infection despite their low sensitivity and the intrinsic delay to obtain the results. Certain biomarkers have the benefit of rapid turnover, potentially providing an advantage in timely diagnosis leading to accurate treatment. Over the last few decades, there is an ongoing quest for the ideal biomarker in sepsis. Procalcitonin (PCT), when used alone or alongside additional clinical information, has shown to be a promising tool to aid in the diagnosis and management of patients with sepsis. In February 2017, the Food and Drug Administration (FDA) approved the use of PCT to guide antibiotic treatment in lower respiratory tract infections and sepsis. Despite a good negative predictive value for bacterial infection, the utility of PCT-guided antibiotic initiation is conflicting at best. On the other hand, the use of PCT-guided antibiotic discontinuation has shown to reduce the duration of antibiotic use, the associated adverse effects, and to decrease the overall mortality.

The current review discusses the history and pathophysiology of procalcitonin, synthesizes its utility in the diagnosis and management of sepsis, highlights its limitations and compares it with other biomarkers in sepsis.

## Introduction

Sepsis, the leading cause of death globally, has an estimated incidence of 18 million cases per year worldwide and a mortality rate of approximately 30%.^1^ The increasing incidence of sepsis^2^, ^3^ and the rising cost of treatment^1^ make accurate diagnosis and aggressive treatment a priority to health care delivery systems. The most important interventions to improve survival in septic patients are the timely and appropriate administration of antibiotics and resuscitation in the initial hours after sepsis develops.^1^, ^3^, ^4^ Early diagnosis is fundamental to prompt initiation of treatment. Sepsis was classically defined as Systemic Inflammatory Response Syndrome (SIRS) caused by documented or suspected microbial infection; however, identification of the organism causing such infection is not always straightforward. Therefore, the differentiation of sepsis from non-infectious SIRS can be very challenging. The time to administration of antibiotics after sepsis identification is recognized as a key performance indicator in the management of sepsis^4^, ^5^; however, inappropriate and unnecessary use of antibiotics can be harmful and is a major cause behind the rising rates of antibiotic resistance.

Culture methods with microbial isolation continue to be the gold standard for diagnosis of infection despite their low sensitivity. Blood cultures are negative in up to two-thirds of cases and cultures from all sites are negative in up to one-third of sepsis cases.^2^ In addition, microbial cultures have an inherent delay for result availability, potentially hindering the implementation of timely and effective interventions. Compared to microbial isolation techniques, biomarkers tend to increase in the early stages of the sepsis, can be instantly tested with a rapid turnaround and show increased levels of expression in sepsis compared to non-infectious SIRS. These characteristics would potentially allow an accurate and timely diagnosis that will lead to prompt treatment. There has been an ongoing search, over the last few decades, for an 'ideal biomarker' in sepsis. This biomarker must possess a high diagnostic accuracy (high sensitivity, specificity, positive predictive value and negative predictive value). Procalcitonin, when used in combination with additional clinical information, has shown to be a promising tool in the diagnosis and management of patients with sepsis.

## The basics of Procalcitonin

The intracellular precursor of calcitonin, currently known as procalcitonin (PCT), was first discovered in 1975 during the study of calcitonin biosynthesis in chicken ultimo-branchial glands.^6^ In 1981, a similar molecule was discovered in human thyroid medullary carcinoma tissue leading to the description of the exact structure of PCT.^7^ The level of PCT in healthy individuals is much lower than the detection threshold and was only known to increase in patients with medullary thyroid and small cell lung carcinoma. Elevated levels of PCT in patients with bacterial infections were reported for the first time in 1993.^8^ Since then, PCT has become an important biomarker and is increasingly being used in the context of sepsis.

**Figure 1:**
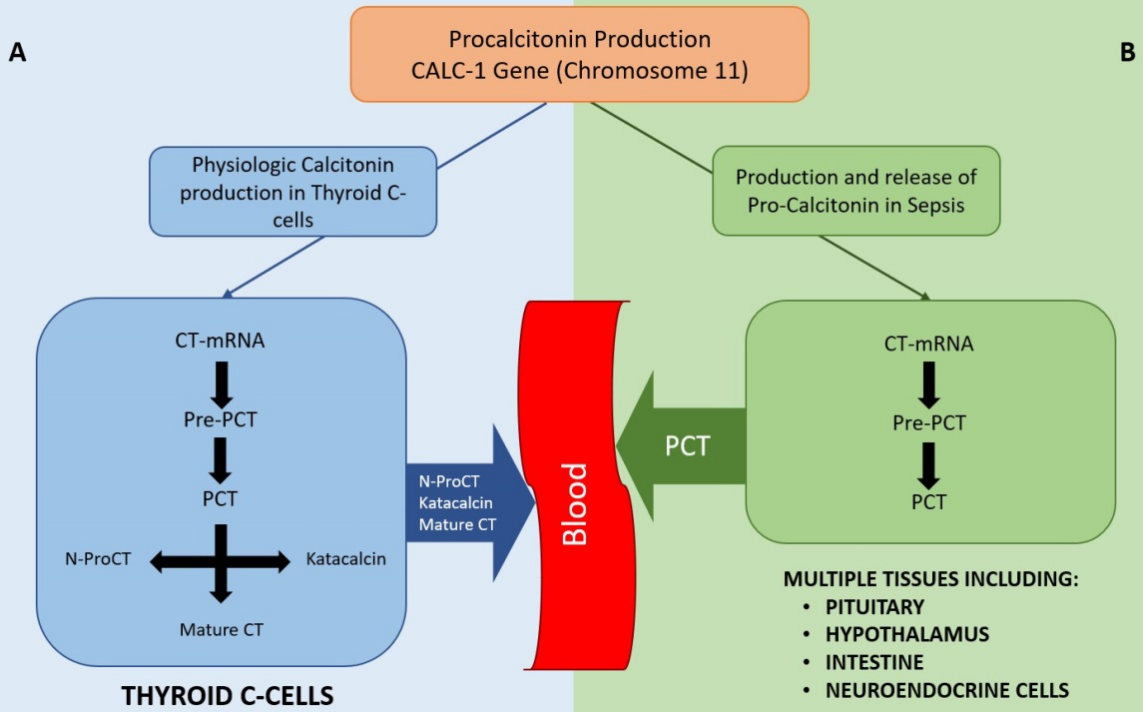
** Diagram showing physiological production of Calcitonin *(A)* and production of PCT in sepsis states *(B)*.** As depicted, PCT is not typically secreted into the bloodstream in normal physiological conditions whereas multiple tissues can secrete PCT in sepsis. Once in the bloodstream, PCT cannot be degraded. CT-mRNA: Calcitonin messenger RNA, Pre-PCT: Pre-procalcitonin, PCT: Pro-calcitonin, N-ProCT: N termical Pro-calcitonin, CT: Calcitonin

In healthy individuals, PCT is produced in the thyroid C-cells, cleaved from pre-procalcitonin by an endopeptidase in the endoplasmic reticulum (Figure 1). PCT is then further broken down to form N-terminal PCT, C-terminal katacalcin and active calcitonin. As all the PCT formed in the C-cells is broken down into the above-mentioned products and no PCT enters the circulation, the serum PCT level in healthy subjects is below detectable level. Moreover, no plasma enzymes are able to breakdown PCT once it enters the circulation; hence, it remains unchanged with a half-life of 25-30 hours.^9^

During inflammation and sepsis, the production of PCT follows an entirely different pathway (Figure 1), details of which are not fully understood. Several studies have shown the production of PCT in response to bacterial Lipopolysaccharide (LPS) or other endotoxins and to inflammatory markers like IL-β, IL-6, TNF-α, IL-2, etc.^8^-^10^ The presence of PCT in the serum of thyroidectomized patients during bacterial infection supports the notion that an organ other than the thyroid is the source of PCT in bacterial sepsis.^8^ Some studies suggest a ubiquitous expression of the calcitonin gene in multiple tissues in response to sepsis^11^, while others suggest that specific organs like pituitary, neuroendocrine cells in the lungs, intestine, splanchnic area, liver or hypothalamus are the source of PCT in sepsis.^12^

## Role of Procalcitonin in the management of sepsis

PCT has been increasingly used in the management of sepsis. Patients with higher PCT levels^13^, ^14^ or those in whom the PCT levels remain elevated for longer duration despite treatment tend to have a worse prognosis compared to the ones with lower levels of PCT or in whom the PCT level decreases soon after initiation of antibiotics.^15^-^18^ While PCT use as a diagnostic tool for sepsis or to decide to initiate antibiotics has been controversial, the evidence supporting PCT use in the discontinuation of antibiotics is stronger.

## PCT as a diagnostic tool

PCT is an intriguing biomarker for the early diagnosis of sepsis in critically ill patients. Several studies have supported the use of PCT for making a diagnosis of sepsis^11^, ^19^-^22^, while others have recommended its use to rule out sepsis.^23^ It has a good negative predictive value for bacterial infection^24^, especially bacteremia^25^-^29^, although the current evidence does not suggest a suitable threshold to exclude bacteremia. PCT is detectable in the serum within a few hours after its induction, reaches its peak within 24 hours, and if treatment is adequate, the levels start to decline by approximately 50% per day.^30^, ^31^ On the other hand, if the treatment is not adequate, PCT remains elevated or rises further. The initial increase in PCT levels is more prominent in bacterial infections than in viral infections or non-infectious SIRS.

A meta-analysis^19^ of 25 studies with 2966 patients concluded that PCT could be used as a quick and early diagnostic test of sepsis in critically ill non-immunocompromised adults. The role of PCT in detecting sepsis in immunocompromised patients, however, remains controversial as studies have shown conflicting results.^32^-^35^ On the other hand, PCT has demonstrated an acceptable accuracy for the diagnosis of bacterial infections in septic patients with liver cirrhosis compared to patients with normal liver function.^36^ These results contrast with those of Tang et al.^37^ who described a low diagnostic performance of PCT in differentiating sepsis from non-infectious SIRS in critically ill adults. This meta-analysis suggested not to use PCT as a diagnostic tool for sepsis. While PCT is not a perfect marker for diagnosis of sepsis, it is thought by some to be one of the most promising tests available.^22^

It is important to note that these studies compared PCT to a clinical diagnosis of sepsis; therefore, the results could be confounded by clinician variation. Moreover, the lack of a uniform cut-off level of PCT used for the diagnosis of sepsis could potentially explain the difference in the stated results. Despite growing evidence supporting the utility of PCT, its use as a diagnostic tool in sepsis remains questionable because of relatively low sensitivity and lack of a precise cut-off level.

## Initiation of antibiotics

A randomized controlled trial looking at PCT guided initiation of antibiotics in critically ill patients did not show effectiveness in decreasing the total antibiotic days.^38^ The recent ProACT trial^39^ failed to show a benefit in terms of mortality or antibiotic exposure for patients with suspected lower respiratory tract infection when PCT was used to guide antibiotic initiation. Pre-specified subgroup analysis, however, showed that the PCT strategy was useful in reducing antibiotic exposure in the emergency department in patients with acute bronchitis but not in other sub-groups like COPD exacerbation, pneumonia and asthma exacerbation. Despite the reduction in emergency department antibiotic exposure in the acute bronchitis sub-group, there was no significant difference in antibiotic use by day 30.

Several studies^40^-^42^ have shown that antibiotic administration based on a PCT algorithm decreased the rate of antibiotic exposure and antibiotic-associated adverse effects without significant difference in clinical outcomes. However, these studies do not differentiate the advantage of incorporating PCT in the decision to initiate antibiotics versus discontinuation of antibiotics. Schuetz showed a significant decrease in antibiotic use when PCT was used to decide on initiation, as well as the discontinuation of antibiotics.^40^ However, Bouadma et al. and Christ-Crain et al. showed that while the difference in antibiotic use was low between the groups on admission and day one, it rose significantly on subsequent days.^41^, ^42^ Interestingly, a meta-analysis evaluating the role of PCT in the initiation of antibiotics^43^ showed a benefit in decreasing the antibiotic exposure compared to standard care with no associated morbidity or mortality benefit. However, this study had the limitation of including only two studies. Overall, the bulk of data suggests that PCT is more useful to guide the decision regarding discontinuation of antibiotics than to decide on their initiation.

The Infectious Diseases Society of America (IDSA) and American Thoracic Society (ATS) guidelines for the treatment of Community-Acquired Pneumonia (CAP)^44^, Ventilator-associated Pneumonia (VAP) and Hospital-acquired pneumonia (HAP)^45^ recommend against using PCT in the decision to initiate antibiotics. The use of PCT to aid in the decision to initiate antibiotics has failed to show benefits in clinical outcomes and its role in decreasing antibiotic exposure and antibiotic-related adverse outcomes is conflicting at best. Based on currently available information, the use of PCT in making the decision to initiate antibiotics is not recommended.

## Discontinuation of antibiotics

As previously discussed, PCT level drops rapidly (50% in 24 hours)^30^, ^46^ once the patient gets adequate treatment. On the contrary, persistently high or up-trending PCT levels suggest that the treatment strategy may not be appropriate. This property of PCT can be used to guide the treatment of sepsis.

PCT guided antibiotic discontinuation did not show a mortality benefit when a low-level PCT cut-off (1 ng/dl) was used.^47^ The low cut-off may have potentially lead to unnecessary antibiotic administration. Moreover, one of the studies in question included a large number of surgical patients (40%) where an elevated PCT level is generally expected. Similarly, the ProACT trial^39^ and the study be Shehabi et al.^48^ failed to show a lower antibiotic use in PCT-guided therapy compared to usual therapy.

Multiple other studies, however, have shown that PCT guided antibiotic protocol reduces cumulative antibiotic exposure in patients^40^-^43^, ^49^-^52^. However, these studies have failed to show a reduction in the risk of antibiotic-related *Clostridium difficile* diarrhea, the emergence of antibiotic resistance, or overall mortality.

De Jong et al.^53^ showed a significant decrease in mortality with the use of PCT-guided therapy in critically ill patients. This was supported by a Cochrane based research^54^, which involved 26 trials including 6708 participants and showed that a PCT-guided strategy on the initiation and duration of antibiotic treatment in acute respiratory infections results in a lower risk of mortality, lower antibiotic consumption, and lower risk for antibiotic-related side effects. Similarly, a recent study by Lam et al.^55^ showed that PCT guided cessation of antibiotics resulted in lower mortality.

The IDSA/ATS guidelines^45^ for treatment of VAP/HAP and the Surviving sepsis campaign guidelines^3^ give a weak recommendation based on low-quality evidence that PCT levels can be used to discontinue empiric antibiotics.

While the mortality benefit of a PCT guided strategy to discontinue antibiotics is questionable, there is enough data to suggest that PCT has shown to decrease antibiotic exposure and antibiotic-related adverse effects. Based on the available data, we feel strongly that PCT can be incorporated into the management of sepsis and used in making the decision to discontinue antibiotics in appropriate patients.

## Comparing PCT to other markers for sepsis

### Presepsin

We have limited data on direct comparison between PCT and presepsin. While PCT has a higher diagnostic accuracy than presepsin^21^, presepsin can signal the changes in the clinical course (improving or worsening sepsis) much faster.

Presepsin has a pooled sensitivity of 0.88, while that of PCT is 0.75. The pooled specificity of presepsin is 0.58 compared to 0.75 for PCT.^56^ The data on presepsin is limited and may not be a readily available test in many facilities.

### C-Reactive Protein

C-Reactive Protein (CRP) is an acute-phase reactant and a sensitive marker for inflammation and tissue injury that cannot differentiate infective from non-infective causes of inflammation. The slow variation of CRP levels constitutes another major limitation compared to PCT. Therefore, an otherwise normal CRP level could introduce an unacceptable delay in starting appropriate antibiotic treatment, ultimately affecting mortality.

Compared to CRP, PCT has a better predictive value for admission to the special care unit and duration of intravenous antibiotic use.^57^ In addition, PCT has a higher sensitivity and specificity, particularly in autoimmune and malignant diseases^58^, ^59^ regardless of the use of corticosteroids or immunosuppressive agents.^60^

## Limitations of PCT

Despite being the most accepted biomarker in sepsis, PCT is far from being an ideal biomarker. PCT has many limitations that should be taken into consideration when incorporating PCT in the management of a potentially septic patient.

Falsely low PCT in localized infections like cellulitis, appendicitis, abscess and empyema can be misleading.^30^, ^61^-^63^ However, some data shows a modest performance of PCT in differentiating para-pneumonic effusion from malignant or transudative effusions.^64^ Interpretation of PCT levels can be difficult in patients with severe trauma, major burns, multi-organ failure, islet cell tumors and medullary thyroid carcinoma. It is also important to acknowledge that cellular injury of any kind, whether direct tissue or ischemia-reperfusion injury without infection, can result in elevation of PCT.^30^ Highly elevated bilirubin^65^ and triglycerides (>1000mg/dl) also interfere with PCT level measurement.

Serum levels of PCT increase in patients with deranged renal function. Considering the high incidence of renal failure in patients admitted to hospital and critical care units, the utility of PCT might be limited. While some studies report that PCT can still be used in patients with renal failure^46^, ^66^ including those on hemodiafiltration^67^, others caution about the need to use a different threshold (2.57 ng/ml instead of 0.8 ng/ml).^68^ Although the cut-off for PCT level might need to be adjusted in patients with different levels of renal function, the presence of kidney injury per se should not preclude the use of PCT. Moreover, the PCT trend could still be used in these patients with renal failure.

## Conclusion

Serum PCT is a promising biomarker for early detection of bacterial sepsis with a good negative predictive value for bacteremia. The current evidence supports PCT-guided discontinuation of antibiotics, while the data behind PCT-guided initiation of antibiotics is not as strong.

The current studies addressing the utility of PCT in sepsis management are conflicting as patient characteristics and clinical settings vary markedly among them. The use of different PCT cut- off levels as well as different algorithms for PCT guidance make the interpretation even more difficult. However, most of the studies have shown that PCT guidance results in lower antibiotic exposure and antibiotic-associated adverse effects without compromising clinical outcomes. Nonetheless, PCT should always be used in conjunction with clinical findings.
